# Food safety policies and practices in public spaces: The urban water, sanitation, and hygiene environment for fresh fish sold from individual vendors in Mzuzu, Malawi

**DOI:** 10.1002/fsn3.1155

**Published:** 2019-08-08

**Authors:** Jazimoni Lazaro, Fanuel Kapute, Rochelle H. Holm

**Affiliations:** ^1^ Department of Fisheries & Aquatic Science Mzuzu University Mzuzu 2 Malawi; ^2^ Centre of Excellence in Water and Sanitation Mzuzu University Mzuzu 2 Malawi

**Keywords:** developing countries, fish, food safety, Malawi, sanitation, urban

## Abstract

In sub‐Saharan Africa, informal markets account for more than 80% of the total food selling. Fish is a major protein source for households in Malawi and is commonly purchased from individual vendors. The aim of this study was to review national acts and policies and local regulations focused on fresh fish sold at open‐air markets or by mobile vendors and to further examine the water, sanitation, and hygiene environment that may impact food safety in Mzuzu City, Malawi. The study used interviews, an observational checklist, and sampling of water and fish skin. In general, there was limited oversight of food safety where fresh fish are sold by vendors, and food safety guidance was inadequate. There was access to water in three of the four markets, but only two markets had safe water (0 cfu/100 ml for *Escherichia coli*). All vendors stored water in a container for use throughout the day to sprinkle over the fish with their bare hands to keep them from drying out. The mean washing water *E. coli* level was 700 cfu/100 ml. All fish skin samples (25/25) were positive for the presence of *Salmonella* spp., and most had high levels of *E. coli*. Sanitation facilities were available for vendors and customers in two of the four markets, but the use was limited. This research identified three key opportunities: (a) Regulatory framework including informal markets and mobile vendors; (b) Safe water, clean and functional toilets, and handwashing stations with soap at every market; and (c) Foodborne disease education for vendors.

## 1 INTRODUCTION

In sub‐Saharan Africa, informal markets account for more than 80% of the total food selling, which play a role in both food security and food safety (Roesel & Grace, 2014). Foodborne diseases, caused by viruses, bacteria, and protozoa from unsafe food, are a global problem with an especially high burden in Africa. Children under 5 years of age are especially vulnerable (World Health Organization [WHO], 2015). Where food security is a concern, food safety is even more important. Malawi is one of the countries in southern Africa where malnutrition among children under the age of 5 is high, with 37% and 3% of children classified as having chronic or acute undernutrition, respectively (National Statistical Office [Malawi] and ICF, 2017). Although poor water supply, sanitation, and hygiene conditions have been linked to a high prevalence of undernutrition (Cumming & Cairncross, 2016; Dodos, Mattern, Lapegue, Altmann, & Aissa, 2017), the focus of previous studies has been on household access rather than where food is sold from urban public spaces. Morse, Masuku, Rippon, and Kubwalo (2018) noted that in Malawi “there is a significant threat to public health and {local food} market access due to uncoordinated, outdated or incomplete regulatory framework, poorly defined mandates, limited infrastructure, lack of equipment and skilled personnel, inadequate resources, and limited awareness and ability to comply with standards.”

Riley, Chilanga, Zuze, and Joynt (2018) noted that in Mzuzu, Malawi, fish is a main source of dietary protein, and fish was rated as more affordable by households than beef, pork, chicken, or other meat. Fresh fish is regularly consumed by 47% of households and is purchased at either area markets or from street sellers (Riley et al., 2018). Fish do not naturally harbor *Escherichia coli* (*E. coli*) or *Salmonella* spp. (Heinitz, Ruble, Wagner, & Tatini, 2000; Hansen, Clark, Ishii, Sadowsky, & Hicks, 2008; Food and Agriculture Organization of the United Nations [FAO], 2012). However, contamination can occur along the supply chain, as fish are handled by several intermediaries from the time of capture to sale at local markets (Samikwa, Kapute, Tembo, Phiri, & Holm, 2019). Heinitz et al. (2000) found a 12.2% incidence of *Salmonella* spp. in fresh fish imported into the United States.

The aim of this study was to review national acts and policies and local regulations focused on fresh fish sold at open‐air markets or by mobile vendors and perform a detailed case study to further examine the water, sanitation, and hygiene infrastructure, vendor knowledge and behavior, and (as applicable) the presence of *E. coli* and *Salmonella* spp. that may impact food safety in Mzuzu City, Malawi.

## 2 MATERIALS AND METHODS

### 2.1 Study area

**Figure 1:**
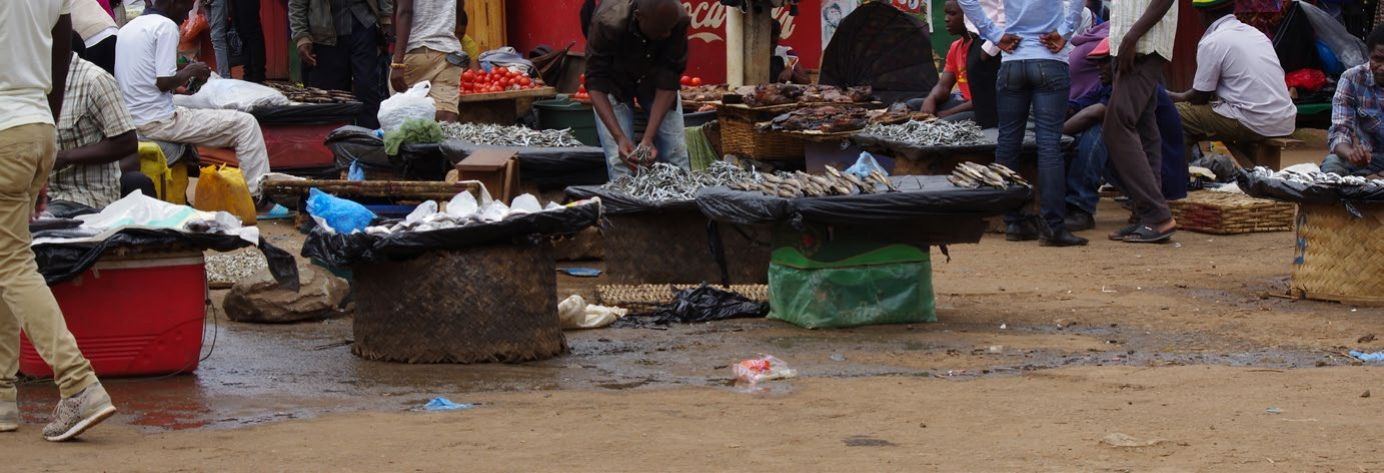
Fresh fish being sold at an open‐air market, Mzuzu, Malawi

The study was conducted in Mzuzu City, Malawi, which covers 146 square kilometers and has a population of 220,000 according to the 2018 census (Malawi Government, 2018). There are 15 open‐air markets in the city, which provide products for daily consumer demands, including local fruits, vegetables, grains, and protein (Figure 1). For 76% of households, a food market can be reached in less than a 30‐min walk (Mzuzu City Council, 2014).

### 2.2 Data collection methods

Study data were collected using an observational infrastructure checklist, interviews, sampling of water, and sampling of fresh fish skin from October 2018 to February 2019.

Following a review of national acts and policies, and local regulations, four agencies found to be present in Mzuzu and able to provide monitoring or evaluation were interviewed using a structured interview in English. They were asked what they knew of the regulatory framework covering water, sanitation, hygiene, and food safety supporting fresh fish sold at markets or by mobile vendors. In addition, they were asked the frequency and type of monitoring and evaluation performed.

Five groups of individual fresh fish vendors were included in our study: four groups operating at open‐air markets (Area 1B Market, Chibavi Market, Mzuzu Central Market, and Zolozolo Market) targeting a range of smaller and larger permanent markets that sell fresh fish and a fifth group of mobile vendors that sell fresh fish in a basket on the back of a bicycle on roads adjacent to the Area 1B Market and Zolozolo Market. Five vendors that sold fresh *Engraulicypris sardella* (usipa) or *Opsaridium microcephalum* (sanjika) were purposively selected from each group (*n* = 25). For vendors at the permanent open‐air markets (*n* = 20), a structured interview was conducted in the local language, Chichewa, which covered vendor behavior impacting food safety, including use of and satisfaction with sanitation facilities at their place of work and their knowledge of food safety. Mobile vendors were not interviewed in our study because they wanted to keep moving for sales.

At each permanent open‐air market (*n* = 4), the use of the nearest sanitation facility (including access for both vendors and customers) was observed, and the same researcher (the first author) counted the number of users who went in/out of the sanitation facility over a period of 1 hr between the hours of 8 a.m. and 4 p.m. for five consecutive days. Immediately following this, the number of general market users (including vendors and customers) was counted over a 1‐hr period. An observational checklist that covered water access, handwashing station with soap, and sanitation facility infrastructure was completed at each market (*n* = 4).

When a water source was available at the market, a 100 ml water sample was collected (*n* = 3; Mzuzu Central Market, Chibavi Market, and Zolozolo Market). Two fresh fish were purchased, and a 100 ml sample of water used for washing the fresh fish was also collected from the 25 vendors. Water samples were collected in Whirl‐Pak^®^ bags containing sodium thiosulfate (Nasco). Fish samples were collected in Whirl‐Pak^®^ plastic bags. Samples were transported and analyzed at an academic laboratory at the department of Fisheries and Aquatic Science, Mzuzu University.

*Escherichia coli* and *Salmonella* spp. were selected as nonindigenous bacteria of public health significance introduced in fish and fishery products through environmental contamination by domestic and/or industrial waste (FAO, 2012). The *E. coli* analysis was performed using a field‐based plate method for contamination monitoring (Thomas, Andrés, Borja‐Vega, & Sturzenegger, 2018). The *Salmonella* spp. analysis methodology was based on Heinitz et al. (2000), but the product sample was reduced to a 1.5 g portion and the procedure adapted for a field‐based method based on prestudy trials in our laboratory.

Water samples (*n* = 28; 25 samples of water used by individual vendors for washing the fresh fish onsite and three market water source samples) and fresh fish skin samples (*n* = 25) were analyzed for *E. coli* using 3M™ Petrifilm™ *E. coli*/Coliform Count Plates (3M™). For market water source samples (*n* = 3), a 100 ml volume was sampled. Fresh fish samples (*n* = 25) were prepared by laterally scraping the skin surface (approximately 1.5 g) using an aseptic blade and adding the skin to 10 ml previously boiled and cooled water in a Whirl‐Pak^®^ plastic bag. The mixture was hand shaken for 2 min, and then, 1 ml of this water was analyzed for *E. coli*.

All samples for *E. coli* analysis were incubated within 6 hr of sample collection. Samples were incubated at 35°C for 24 hr, and the results are presented as colony forming units per 100 ml (cfu/100 ml) for water and cfu/g for fish skin. An equipment blank, as a negative control, of boiled and cooled water was analyzed each day (*n* = 5), and a value of zero for *E. coli* was found for each. No positive controls for *E. coli* or total coliforms were conducted.

Additionally, for fresh fish skin samples (*n* = 25), *Salmonella* spp. was analyzed using the 3M™ Petrifilm™ Salmonella Express system (3M™). A second fish sample was prepared by combining approximately 1.5 g of skin and 10 ml of prepared 3M™ Salmonella Enrichment Base and 3M™ Salmonella Enrichment Supplement in a Whirl‐Pak^®^ plastic bag. The mixture was incubated at 41.5°C for 24 hr. Then, a cotton swab was dipped in the sample, and it was spread on a 3M™ Petrifilm™ Salmonella Express Plate. Samples were incubated a second time at 41.5°C for 24 hr, and presumptive positives were identified. Five equipment blanks using boiled and cooled water, but the same methodology, were analyzed as a negative control, and all were found to be negative for *Salmonella* spp. No positive controls for *Salmonella* spp. were conducted.

### 2.3 Data analysis

Laboratory samples were analyzed in duplicate, and the mean results were recorded. Statistical analyses were conducted using the R Project 3.5.1 statistical package (Vienna, Austria). Water source results for *E. coli* were compared to national and WHO guidelines for drinking water (Malawi Bureau of Standards [MBS], 2013; WHO, 2017). If a *p* value was less than a .05 significance level, we concluded there was a significant difference.

### 2.4 Ethics

This study and its informed consent procedures were approved by the Republic of Malawi National Commission for Science and Technology (Protocol Number P.10/18/327). Informed, verbal consent was obtained from the study participants.

## 3 RESULTS

### 3.1 National acts, policies, and local regulations

The key national acts, policies, and local regulations covering water, sanitation, and hygiene infrastructure standards, and food safety evaluation criteria for fresh fish being sold at urban open‐air markets or by mobile vendors are presented in Table 1. While some of these mention markets, none address mobile vendors. There were no specific criteria in national acts or policies regarding standards for water, sanitation, and hygiene infrastructure, or monitoring and evaluation guidelines in public spaces where fresh fish are being sold. There were, however, some local regulations (Mzuzu City Assembly, 2001), but with limited monitoring and evaluation criteria.

**Table 1:** Regulatory framework supporting the link between water, sanitation, and hygiene infrastructure standards and food safety evaluation criteria where fresh fish are sold, Mzuzu, Malawi

Title	Date	Relation and gaps
Public Health Act	1968	Focus on food intended for human consumption, including sanitary control of food‐selling premises. No specific guidance on how to monitor or evaluate. Generally, an out‐of‐date policy developed soon after independence.
Local Government Act	1998	City councils have control to establish, maintain, and manage places where food is sold at markets, market buildings, and surrounding premises.
Mzuzu City Food by‐laws	2001	Fresh fish should only be sold at market places or other authorized premises. Fish should be covered and protected from contamination when in transit. States, in limited detail, that water and sanitation facilities must be available at food‐selling premises but does not include criteria for the required number of or standards for available water sources or sanitation facilities that need to be available. Does not include limits for coliforms or *Salmonella* spp. in food.
Malawi Bureau of Standards Food and food processing units code of hygienic conditions	2002	Focus on the growing, harvesting, preparation, processing, packaging, storage, transportation, distribution, and sale of food but with no criteria for informal food‐selling premises such as markets or mobile vendors, or fish sales.
National Water Policy	2005	States that water and sanitation facilities should be available for both customers and vendors at urban market areas but does not include criteria for the required number of or standards for water sources or sanitation facilities that need to be available.
Food Security Policy	2006	Reference to fish is per sustainable food production. Limited link between food safety and food security.
Malawi Bureau of Standards Fresh fish specification	2007	Specific guidance criteria cover the handling, preparation, distribution, and packaging of fresh fish with a focus on large retail (permanent) operators. There are no guiding criteria on fish sales for informal food‐selling premises such as markets or mobile vendors. Does not include limits for coliforms or *Salmonella* spp. in food.
National Sanitation Policy	2008	Recommends market traders manage sanitation through committees for solid waste disposal, recycling, and public sanitation facilities. There are no criteria for the required number of or standards for handwashing stations or sanitation facilities that need to be available per market or user population in urban areas.
Malawi Bureau of Standards Drinking water specification	2013	States physical, organoleptic, chemical, and microbiological limits for consumed piped water.
National Fisheries and Aquaculture Policy	2016	Focus on fish quality and value addition but no specific guidance on how to monitor or evaluate food safety for fresh fish.
National Environmental Act	2017	Focus on public participation in environmental management but no reference to environmental planning specific to public spaces where food sellers operate.

The oversight of national acts and policies and local regulations for water, sanitation, hygiene, and food safety covering fresh fish sellers operating in Mzuzu City falls under four agencies: (a) Ministry of Health; (b) Department of Fisheries under the Ministry of Agriculture, Irrigation, and Water Development; (c) MBS; and (d) Mzuzu City Council. We did not consider issues related to labor conditions in our study.

The Ministry of Health respondent described the difficulty in providing oversight of fresh fish sellers as follows (female interviewee, 11 February 2019):

There's currently no specific food safety policy governing the sale of fresh fish by mobile vendors. But in general terms, the Public Health Act gives powers to health inspectors to inspect food and food premises to ensure that food is wholesome and safe for human consumption. Monitoring of this has worked in formal food premises but has been haphazard in the informal food sector as we do not have policies that specifically address informal food markets. Existing WASH {water, sanitation, and hygiene} policies do not address this issue either. Monitoring in this context is mainly visual.

The Department of Fisheries under the Ministry of Agriculture, Irrigation, and Water Development commented as follows on the gaps in oversight checkpoints (female interviewee, 5 February 2019):

As {the} Mzuzu Regional Office, our mandate is to issue sanitary certificates to traders who have bought fish products from markets within the northern region. Inspection is visual only specifically; we look for any damages due to fungal or handling damage; sometimes {using the} olfactory sense as a measure of the quality of the fish. Laboratory analysis is not possible because of {the} lack of laboratory equipment. The sanitary certificates are issued to those traders exporting the fish outside Malawi. The office of the District Fisheries is mandated to inspect {the} quality of fish that have been landed on the beach only.

The Department of Fisheries has a regional office in Mzuzu, but there is no daily presence of staff to monitor the fresh fish markets within the city.

The MBS responded (male interviewee, 8 February 2019) as follows:

{The} Malawi Bureau of Standards monitors fresh fish processing done by formal businesses who add value to the fish. Monitoring of fresh fish sold at the open‐air markets or by mobile vendors is done by city councils and the Department of Fisheries. Value addition to fresh fish includes further processing and/or packaging. All traders in Malawi doing value addition onto fresh fish are monitored by the MBS using the following standards:

- Frozen Fish – Specification. MS 115:2002
- Food and food processing units code of hygienic conditions, first revision. MS 21:2002
- Labeling of prepacked foods – General standard. MS 19:2001.

The respondent from the MBS knew there was a frozen fish standard but was not aware of the fresh fish specification from their same organization (MBS, 2007).

The Mzuzu City Council responded (male interviewee, 8 February 2019) as follows when asked about their monitoring of fresh fish vendors:

Mostly visual or fish smell too, but when suspicious, {a} microbial {laboratory} test is done. Laboratory analysis of water and food samples is done at the central laboratory in Lilongwe. Monitoring is done routinely by health officials, but a joint team conducts inspection{s} every 3 months. Environmental Health Officers conduct the monitoring in conjunction with {the} MBS, labor office, and industry and trade officers.

While the Mzuzu City Council had the highest level of awareness about national acts and policies and local regulations, their monitoring and evaluation practices that were conducted were not consistent from those of other respondents.

### 3.2 Water, sanitation, and hygiene environment for fresh fish vendors

**Figure 2:**
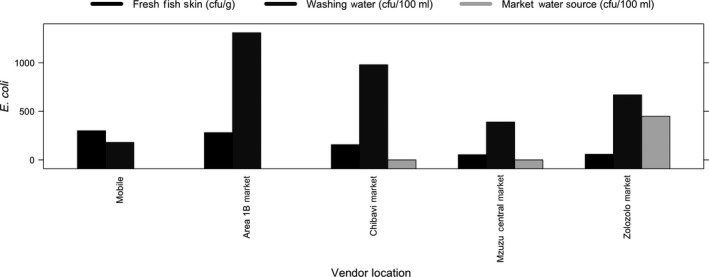
*Escherichia coli* in fresh fish skin, stored washing water, and water sources where fresh fish are sold, Mzuzu, Malawi

Mzuzu City Assembly (2001) regulations note that piped water should be available at food‐selling premises. Only three of the four markets had any water access for vendors. Of these, the two markets with piped water (Chibavi Market and Mzuzu Central Market) had safe water (0 cfu/100 ml for *E. coli* and safe based on MBS, 2013 and WHO, 2017), whereas the Zolozolo Market was using a shallow well which had *E. coli* levels of 450 cfu/100 ml (Figure 2).

When water was available, the market vendors (15/15) reported using it; however, at the one market without a water supply (Area 1B Market), vendors reported either obtaining water from their home (4/5) or buying it from a nearby shop (1/5).

All vendors (25/25) used stored onsite water to sprinkle over the fish throughout the day to keep them from drying out; they called this “washing water.” Mobile vendors stored water in a 1‐ or 2‐L plastic bottle. Market‐based vendors stored it in a 5‐ to 20‐L metal or plastic bucket. Water was splashed from the water storage container onto the fish with bare hands.

The mean *E. coli* level for washing water was 700 cfu/100 ml, and the mean level for fish skin was 170 cfu/g. All samples of fish skin (25/25) were positive for the presence of *Salmonella* spp. A Mann–Whitney test comparing the median *E. coli* level in washing water used by mobile vendors with that in washing water used by market‐based vendors revealed no difference (*p* = .052), though many (20/25) samples had *E. coli*. A Mann–Whitney test comparing *E. coli* levels on fish skin from mobile vendors with that on fish skin from market‐based vendors also revealed no difference (*p* = .062), though all samples had *E. coli*. All market‐based vendors (20/20) indicated that drinking water during their work day was available to purchase, but this water was not analyzed for *E. coli* in our study.

**Figure 3:**
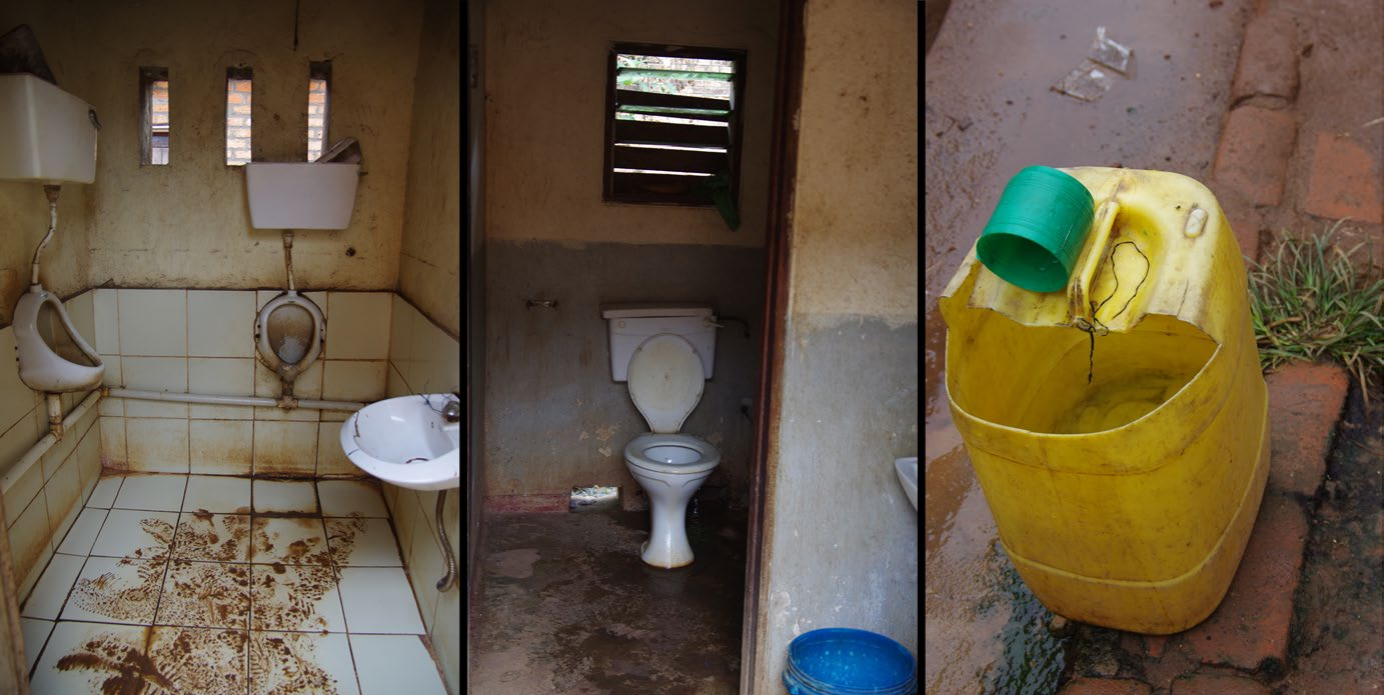
Urinal, flush toilet, and handwashing station infrastructure near Mzuzu Central Market fish vendors

Fresh fish vendors at each market were clustered together, but the selling sites were adjacent to those for other food items, such as fresh fruits and vegetables. Market areas where fish are sold were observed to be used by 27–69 people per hour, and the Mzuzu Central Market was the busiest with a mean of 54 people per hour. The market vendors reported selling fish at their respective markets for a mean of 6 hr per day (*n* = 20). However, the vendors operating in the Mzuzu Central Market (*n* = 5) had the highest mean at 11 hr per day. Only two markets (Chibavi Market and Mzuzu Central Market) had working sanitation facilities (pour flush, urinal, or a room containing a flush toilet piped to a septic tank) for customers and vendors, and although both had a handwashing station with water, there was no soap present (Table 2 and Figure 3). One other market, Area 1B, had some sanitation infrastructure, but there was a plumbing blockage at the time of data collection, rendering it nonoperational. Where there were sanitation facilities present, the vendors (8/10) generally reported using them, though this was contrary to our researcher observations of a mean of one person per hour using each sanitation facility. The sanitation facilities were designed for a higher level of use than was observed, as one market had five stalls and the other had two stalls.

**Table 2:** Water, sanitation, and hygiene infrastructure where fresh fish are sold at open‐air markets, Mzuzu, Malawi (*n* = 4)

Location	Sanitation										Hygiene		Water
	Cleaned by	Cost per use	Total number of sanitation facility stalls present	Materials for sanitation facility walls	Does each stall have a door for privacy?	Does each door have a lock for security?	Is there visible fecal material in and around the floor or seat?	How is the odor?	Distance from the fish selling site to sanitation facilities (m)	Is a trash can present in the sanitation facility?	Distance from the food‐selling site to handwashing station (m)	Is soap available at the handwashing station?	Is piped water available?
Area 1B Market	No functioning facility available												No
Chibavi Market	Community	MK50	5	Local clay bricks	Yes	Yes	No	Good	17	No	21	No	Yes
Mzuzu Central Market	Council	MK100	2	Local clay bricks	Yes	No	Yes	Bad	27	No	15	No	Yes
Zolozolo Market	No functioning facility available												No

The eight vendors who reported using their market's sanitation facilities said they paid MK50–100 (USD 0.07–0.14) as a pay‐per‐use system. These fees may be a barrier. In order to pay for the use of the sanitation facilities, a vendor in this study would have to sell a small pile of *E. sardella* containing 4–8 fishes or one individual *O. microcephalus*.

The Chibavi market sanitation facilities were cleaned by the community and had the lowest cost per use. Furthermore, all of these vendors (5/5) indicated that they were being managed well and they were satisfied with the safety and cleanliness. It was stated that cleaning was performed daily. In contrast, the second market with a sanitation facility, the Mzuzu Central Market, was cleaned by local government employees (from the City Council) and had fewer (2/5) vendors who indicated that it was being managed well and fewer (2/5) vendors who were satisfied with the safety and cleanliness. When vendors were asked “How does the condition of the sanitary facility present here compare to your home?”, all of the Chibavi market vendors (5/5) reported that it was better than that of their home but fewer of those (2/5) at the Mzuzu Central Market said it was better.

Solid waste was also a problem at each market area, with all market‐based vendors (20/20) indicating that trash was thrown either “everywhere” or in an open pit within the market area, and no market‐based vendors (0/20) noted any waste reduction, reuse, or recycling measures in place at their market.

About half (11/20) of market‐based vendors had heard about foodborne diseases and, when further probed, listed cholera, diarrhea, and (though not appropriate) cough as examples. When asked about symptoms of foodborne diseases, fever, diarrhea, stomach pain, and vomiting were listed. When asked “Do you know how microorganisms that cause foodborne diseases are transmitted?”, 75% of market‐based vendors did not know how microorganisms that cause foodborne diseases were transmitted. The most common disease vendors mentioned that could be transmitted in fresh fish was ringworm (3/20), with one of these respondents stating “stop consuming fish species that cause the disease” as a way to control ringworm. Ringworm though is a skin infection and not a foodborne disease. This indicates a generally low level of knowledge of foodborne diseases or transmission by the vendors in our study.

Many vendors (18/25) sourced their fish from an intermediary. The notable exception was that all vendors (5/5) operating at the largest market, Mzuzu Central Market, obtained their fish directly from Lake Malawi fishers. All mobile vendors (5/5) bought fish from an intermediary. The vendors used bicycles and public transport (local minibuses and taxis) to transport fish; in no case was a vehicle dedicated for food transport reported to be used.

National standards dictate that the holding temperature of fresh fish be maintained as close as possible to 0°C (MBS, 2007), but only three vendors (3/25), all operating at the Mzuzu Central Market, used ice and not necessarily enough to keep all fish at a consistent temperature. No vendors actually monitored the temperature; no thermometers were present or used by vendors. When using ice, vendors reported getting it from a shop within the market area; they did not make their own ice. For the mobile vendors, fish were not covered with block ice, a sunlight barrier, or a dust barrier.

## 4 DISCUSSION

Handwashing with soap and water by vendors while at work and keeping food at a proper temperature appear to be the most effective ways of reducing *E. coli* and *Salmonella* spp. contamination in fresh fish sold by vendors in Mzuzu, Malawi. Still, knowledge of foodborne diseases is low among vendors. To accelerate these behaviors, more training is needed along with proper infrastructure to facilitate the practice. Although the vendors' handwashing practices were not directly observed, the lack of soap was inferred to contribute to the presence of *E. coli* and *Salmonella* spp., as the vendors sprinkled water onto the fish throughout the day. While the FAO (2009) guidelines state that potable water is required when there is contact with fish, only three of the four markets had water available for fish vendors, and this water was potable at only two markets. Local (Mzuzu City Council, 2014) and national (National Statistical Office [Malawi] and ICF, 2017) documents track the water supply and sanitation infrastructure for households, but data are limited for public spaces and mainly comprise data from schools and health facilities.

In our study, we found that the national food safety acts and policies and local regulations for fresh fish that are in place are more applicable to formal retail settings, which primarily serve higher income customers, than to open‐air markets. The largest gap was for mobile vendors, where deficiencies are not being addressed at all. Even for the limited national guidelines that are in place, we found that they were not followed. For example, none of the vendors were observed to have any way of determining whether they were in compliance with holding temperature (MBS, 2007). Many of the findings in our case study agree with those of the nationwide study by Morse et al. (2018): lack of knowledge on national acts and policies and local regulations and standards and that it was not clear who was to perform onsite monitoring and evaluation, frequency of food safety and quality monitoring, or what criteria to use. Pertinently, our study results go further, as the *E. coli* and *Salmonella* spp. data demonstrate that there are problems. Even in the United States, Heinitz et al. (2000) found some incidence of *Salmonella* spp. in fish imported into the United States from around the world, but the findings that all of our fish samples had *Salmonella* spp. and no visible signs of spoilage indicate a need for critical control points to improve food safety in Malawi.

Globally, as shown by Cumming and Cairncross (2016), poor water, sanitation, and hygiene access have in some cases been linked to poor childhood growth and development. Despite these findings, this is a complex issue because interventions in low‐ and middle‐income countries have dominantly focused on household access rather than public spaces where food is present and purchased. For weaning children, even the safety of cooked foods is important. There is an important link between our study results and the work performed by Touré, Coulibaly, Arby, Maiga, and Cairncross (2013) in Mali on home‐prepared soup cooked with local fresh fish and vegetables commonly eaten as a weaning food; they reported that after cooking, 68% of fish soup samples had greater than 100 thermotolerant coliforms/g and that after further storage at room temperature, 90% of fish soup samples had greater than 100 thermotolerant coliforms/g. However, the prevalence of thermotolerant coliforms was effectively reduced after the mothers improved their hygiene practices during cooking and storage by using potable water, washing dishes, washing hands, and reheating leftovers. Importantly, Touré et al. (2013) did not look at reducing fecal contamination in local fresh fish used as an ingredient in the soup at the time of purchase and before it entered the home being linked to the children's health indicators.

While sanitation facilities were only available at two of the four open‐air markets, not all vendors reported using them even when they were present. Vendors pay a daily fee (a tax) to the Mzuzu City Council. For the last reported period, the council recorded that MK40,019,849 (USD 148,000 assuming MK268/USD 1 in 2012) was collected in 2011/2012 (Mzuzu City Council, 2014). The Mzuzu City Council also has several implementing partners working on water supply, sanitation, and hygiene promotion in public spaces. One specific example is the European Union, which invested approximately €3,000,000 (USD 3,400,000) in Mzuzu City between 2013 and 2017 (European Union, 2019). Some of these investments included construction of privately run sanitation facilities in market places and the expansion of the piped water supply:

1. Improving water supply, sanitation, and hygiene promotion in peri‐urban areas of Mzuzu and Karonga Towns. Cost €923,005 from April 2014 to September 2017 by UN Habitat;
2. Integrated WASH intervention in low‐income areas in Mzuzu and Karonga. Cost €1,213,100 from May 2014 to March 2017 by Malawi Red Cross Society through the Netherlands Red Cross; and
3. Peri‐urban sanitation and hygiene project in Mzuzu City. Cost €1,246,907 from December 2013 to May 2017 by Plan Malawi.

The timing of these projects provides an opportunity to envision a food safety chain as part of a comprehensive urban planning approach that combines safe water together with sanitation access for public places, including food markets. The results of our study raise the question of whether these projects actually improved the living standards in urban public spaces or improved food safety for the people of Mzuzu City. For just these three projects, the total investment in Mzuzu was about USD 15 per person in the city. Though the Mzuzu Central Market had new sanitation facilities that were built under one of these projects, the existing facilities adjacent to the fish market included in our study were still being used because of the convenience of being within 30 m of the fish vendors' selling point, as opposed to the new sanitation facility 160 m away and across a busy highway (the newer facility was not included in our study because it was not commonly used by fresh fish vendors). In addition to sanitation facilities being in good condition, a vendor or customer must also want to use them.

Similar to the findings by Afacan and Gurel (2015) on public sanitation facilities in Turkey, our sanitation facilities had issues with cleanliness. However, in our study, even when payment was required to use public sanitation facilities, it did not necessarily mean they were clean. Based on their reported working hours, vendors would likely need to use a sanitation facility multiple times during the day, incurring a cost of MK 50–100 (USD 0.07–0.14) for each use. Thus, sanitation facilities that are not in working order or that people prefer not to use may lead to increased open urination or defecation or the use of other unimproved facilities that are farther away from their place of work. The situation is worse for the mobile vendors who would need to find sanitation facilities on their route and leave their bike and merchandise. The lack of handwashing with soap in all the markets is a critical gap from a food safety standpoint. Based on our study results, at least for the two markets with working sanitation facilities, the sanitation facilities were probably sufficient for users, whereas at the two other markets, this infrastructure should be immediately prioritized. McGinnis, Marini, Amatya, and Murphy (2019) noted that well‐maintained privately owned, pay‐per‐use community sanitation facilities in Nepal were as clean or cleaner than household sanitation facilities. Based on the swab sample results for bacterial concentrations in that study, in community sanitation facilities, the seat/floor, tap/handle/bucket used for anal cleansing, and door handles were the surfaces to focus on when cleaning.

## 5 LIMITATIONS

The number of markets and vendors included in this study was small but did include a range of geographic areas and market sizes. This study did not involve market owners and operators nor did it consider the profit margin of vendors compared to food safety practices or consumer food safety awareness. Intermediaries as a source of introducing contamination into the fresh fish supply chain were not addressed in this study. The fish in this study would traditionally be cooked prior to consumption. Further research to include more of the mobile fresh fish sellers operating in the city is needed.

## 6 CONCLUSION

In conclusion, there are food safety gaps for fresh fish sold from individual vendors in Mzuzu, Malawi, resulting from urban water, sanitation, and hygiene infrastructure, the lack of vendor knowledge and behavior, and inadequate oversight. This study on four open‐air markets and mobile fish vendors found high *E. coli* levels in the water used for washing the fresh fish onsite and on the fish skin, and only two markets had a water source free of *E. coli* for vendors to use. By using *E. coli* and *Salmonella* spp. analyses alongside sanitary surveys and interviews, our study provides some preliminary evidence that indicates a need for critical control points to improve food safety in Malawi despite there being no visible or olfactory signs of spoilage in the fish samples sold by vendors. In general, limited monitoring and evaluation oversight was being performed where fresh fish are sold by individual vendors, and regulator guidance was inadequate. The vendors had a low food safety awareness, yet they spend long hours selling fish and fish are an important source of dietary protein in households. More research is needed in low‐ and middle‐income countries to better understand urban public spaces where food is sold from informal markets and the prevention of malnutrition among urban children under the age of 5 through food safety approaches. This research identified the following three key opportunities for reform: (a) National acts and policies and local regulations must consider informal markets and mobile vendors; (b) Safe water, clean and functional sanitation facilities, and handwashing stations with soap accessible for all food vendors; and (c) Education for food vendors on foodborne disease.

## CONFLICT OF INTEREST

The authors declare that they do not have any conflict of interest.

## ETHICAL APPROVAL

This study was approved by the Republic of Malawi National Commission for Science and Technology (Protocol Number P.10/18/327).

## INFORMED CONSENT

Verbal informed consent was obtained from all study participants.
